# Smoking Status, Body Mass Index, Health-Related Quality of Life, and Acceptance of Life With Illness in Stable Outpatients With COPD

**DOI:** 10.3389/fpsyg.2019.01526

**Published:** 2019-07-02

**Authors:** Kinga Wytrychiewicz, Daniel Pankowski, Konrad Janowski, Kamilla Bargiel-Matusiewicz, Jacek Dąbrowski, Andrzej M. Fal

**Affiliations:** ^1^Faculty of Psychology, University of Warsaw, Warsaw, Poland; ^2^Faculty of Psychology, University of Economics and Human Sciences in Warsaw, Warsaw, Poland; ^3^Clinical Department of Internal Medicine and Allergology, Central Clinical Hospital of the Ministry of Interior in Warsaw, Warsaw, Poland; ^4^Collegium Medicum, Cardinal Stefan Wyszyński University, Warsaw, Poland

**Keywords:** smoking status, obesity, BMI, health-related quality of life, acceptance of life with illness, COPD

## Abstract

Smoking and obesity are important factors related to the etiology and clinical features of chronic obstructive pulmonary disease (COPD). The aim of this study was to carry out deeper analyses of the links between smoking status and body mass index as well as of the links between individual dimensions of health-related quality of life (QoL) and acceptance of life with disease in patients with COPD. Information about BMI, smoking status, clinical features of COPD, a COPD assessment test, and airflow obstruction indicators were obtained from 105 outpatients attending a pulmonary clinic. Analysis of covariance demonstrated that the subgroups of patients distinguished based on smoking status and BMI differed significantly in severity of Cough, Dyspnea, Limitation of daily activities, Lack of self-confidence, and Quality of sleep, independently of sociodemographic factors and clinical features of COPD. The results of our study suggest a certain path of the potential effects of obesity and smoking status on QoL. Risky health behaviors, such as smoking and at-risk body mass, only affect some aspects of health-related QoL.

## Introduction

Chronic obstructive pulmonary disease (COPD) is a chronic disorder that encompasses chronic bronchitis and emphysema; it is characterized by airflow obstruction that is not fully reversible and results in symptoms such as chronic cough, tightness of chest, and shortness of breath (Global Initiative for Chronic Obstructive Lung Disease, 2018).

Data published by Lozano et al. (2012) suggest that COPD is the fourth leading cause of death worldwide, and projections indicate that it will become the third leading cause by 2020 (Mathers and Loncar, 2006) due to the continued prevalence of COPD risk factors and aging populations.

Cigarette smoking is among the most important known risk factors for COPD (e.g., Rimington, 1971) and is estimated to account for 69% of the global burden of this disease (Ezzati et al., 2003). Furthermore, studies have demonstrated that cessation of smoking slows disease progression and improves survival in COPD patients (Godtfredsen et al., 2008). In contrast, ambiguous data exists with respect to the role of obesity in COPD. Some evidence suggests that obesity may have a beneficial effect on the clinical course of COPD. For instance, being overweight or obese decreased the risk of death from COPD (Franssen et al., 2008) whereas being underweight, especially in those with advanced COPD, was associated with increased mortality (Wilson et al., 1989). On the other hand, obesity in COPD patients was also linked to such adverse factors as decreased thoracic compliance, increased airflow resistance, and increased effort breathing, which can worsen symptoms of dyspnea and wheezing due to airflow obstruction (Naimark and Cherniack, 1960). High BMI was also associated with lowered health-related quality of life (QoL) and increased physical limitations due to respiratory symptoms (Arterburn et al., 2004).

The exact nature of the relationships between smoking and body mass, on one hand, and QoL, on the other, is still unclear. QoL in COPD is usually measured by the COPD Assessment Test (CAT) – a questionnaire which combines questions pertaining to both severity of subjective symptoms, such as cough or dyspnea, and more psychologically oriented dimensions such self-confidence or everyday-life limitations due to COPD. The global score on this measure gives an approximation of both severity of symptoms and their impact on functioning. However, when the global score of QoL is taken into account we lose the ability to unravel which dimensions of QoL are indeed affected by other variables, such as smoking or body mass. Therefore, in our study we decided to analyze not only the global index of QoL, but also its dimensions.

Acceptance of life with the disease is a psychological concept related to QoL. Acceptance is defined as a process which takes place from moment to moment, involving active engagement with private events which occur in a given moment without unnecessary efforts to change their frequency or form, especially when such efforts would cause psychological damage (Fletcher and Hayes, 2005). Therefore, with respect to illness, acceptance means internal consent to experience the limitations and unpleasant feelings associated with the disease. Such consent allows a person to engage in activities which are in accordance with their values to achieve their goals despite the limitations of the illness. As such, acceptance of life with illness can be regarded as an important indicator of QoL.

When exploring how smoking and body mass affect QoL in COPD patients, it should be remembered that smoking or body mass may be in covariance with other sociodemographic variables, such as sex or age, and which themselves may be crucial in determining QoL. When calculating simple correlations between smoking and body mass variables, the results may therefore be obscured by uncontrolled interactions between them and other sociodemographic variables. Therefore, we made efforts to control for these potential effects in our analyses.

The aim of this study was to conduct more thorough analyses of the links between smoking status and body mass index, on the one hand, and individual dimensions of QoL in patients with COPD, on the other. To elucidate the effects of these variables on QoL, we decided to carry out analyses which enabled us to clear these relationships of potential confounding factors such as age, gender, disease severity, educational status, marital status, etc.

## Materials and Methods

### Questionnaires

The CAT is a questionnaire for patients with COPD developed by a global team of experts in the field of pulmonology (Jones et al., 2009). It is intended to measure the impact of the disease on a patient’s life and to assess how it changes over time (Papaioannou et al., 2014). The patient assesses eight aspects of illness potentially affecting QoL (Cough, Expectoration, Chest tightness, Dyspnea, Limitation of daily activities, Lack of self-confidence, Quality of sleep, and Energy levels) on a scale ranging from 0 to 5, with higher scores reflecting lower QoL.

The Acceptance of Life with the Disease Scale (ALDS; Janowski et al., 2012) is a self-report questionnaire used to measure the degree of acceptance of one’s life with a disease. It consists of 20 test items, divided into three subscales: (1) Satisfaction with Life Despite the Disease – which measures the sense of contentment, fulfillment, and self-realization in spite of the illness (Cronbach’s alpha = 0.88); (2) Reconcilement with the Disease – the degree of consent with the fact that one has an illness (Cronbach’s alpha = 0.85); and (3) Self-distancing from the Disease – measuring cognitive decentration, the ability to engage in various activities that minimize the importance of the disease (Cronbach’s alpha = 0.72). A global score can be calculated, which is the sum of the scores obtained for all items (Cronbach’s alpha = 0.93).

In the sociodemographic questionnaire, participants provided information about their sex, age, marital status, place of residence, education, and professional activity.

### Clinical Data

The clinical course of COPD: a structured questionnaire was used in which participants provided information on years since COPD diagnosis, number of hospitalizations due to COPD, and made a subjective self-assessment of COPD severity (on a 7-point scale ranging from “very mild” to “very severe”) and progression (on a 4-point scale ranging from “it got a lot worse”; “it got a little worse”; “neither it has deteriorated and improved”; to “it has improved”).

Functional examination of the respiratory system (spirometry) carried out using a MES LUNGEST 1000 spirometer (Krakow, Poland). The following indicators were analyzed: (1) vital capacity (VC), (2) % forced expiratory volume in 1 s (FEV_1%_), and (3) the Tiffeneau-Pinelli index – FEV_1%_/VC_max_.

Smoking status was determined according to the principles set out by the Health Survey for England (HSE; Marston et al., 2014). The participants were divided into three subgroups: never-smokers, ex-smokers, and current smokers. A structured questionnaire was used to collect data on current and past smoking habits, including number of years smoking, number of cigarettes smoked per day, and time elapsed since quitting smoking. Two patients did not provide information about smoking status.

### BMI and Obesity

BMI is a simple index based on weight and height that is commonly used to classify adults as underweight, “normal-weight,” overweight, or obese. It is defined as weight in kilograms divided by the square of height in meters (kg/m^2^). A BMI of 30 or more is widely accepted as denoting obesity (World Health Organization [WHO], 2000).

### Statistical Analyses

Differences between the groups with various smoking statuses and BMI were analyzed using analysis of covariance (ANCOVA), with sex, age, marital status, place of residence, education, professional activity, years since COPD diagnosis, number of hospitalizations due to COPD, subjective self-assessment of COPD severity and progression, and airflow obstruction indicators (FEV_1%_, VC, FEV_1%_/VC_max_) as covariates. Relationships between ALDS and CAT were examined using partial correlations analyses with sex, age, marital status, place of residence, education, professional activity, years since COPD diagnosis, number of hospitalizations due to COPD, subjective self-assessment of COPD severity and progression, and airflow obstruction indicators (FEV_1%_, VC, FEV_1%_/VC_max_) as controlled variables. All statistical analyses were conducted using IBM SPSS statistics version 24.

### Ethical Approval

This study was conducted according to the guidelines of the Declaration of Helsinki. The approval of the Bioethical Commission of the University of Economics and Human Sciences in Warsaw was obtained for this study and all subjects gave written informed consent prior to their participation in the study.

### Participants

The inclusion criterion was stable COPD, as confirmed by a physician; a stable state was operationalized as there being no need to introduce new treatment or change the current treatment. Patients were excluded from the study if their somatic condition related to COPD or other co-morbidities was too severe to enable them to fill in questionnaires. The exclusion criteria were co-occurring mental or neurodegenerative disorders (evidenced in the patient’s medical history) and refusal to give informed consent. All patients were receiving steroid treatment in inhaled form. All participants gave informed consent for participation in the study.

The study sample consisted of 105 out-patients with a diagnosis of COPD, as confirmed by the pulmonologist. The sample included 58 women and 47 men, with a mean age of 70.3 (SD = 9.25) years. Ages ranged from 41 to 91 years. The mean time elapsed since diagnosis of COPD was 10.14 (SD = 9.41) years, and this ranged from 0.25 to 42 years. The mean number of hospitalizations since diagnosis of COPD was 1.11 (SD = 3.31).

The severity of pulmonary obstruction was assessed in accordance with GOLD guidelines. Forty four patients had a grade I obstruction (FEV_1%_> 80%), 43 – grade II [FEV_1%_ ∈ (50%; 80%>)], and 18 patients – grade III [FEV_1%_ ∈ (30%; 50%>)]. The FEV_1%_ index ranged from 29 to 157% (*M* = 74.95%, SD = 24.36%). VC values ranged from 39 to 137 (*M* = 86.88, SD = 21.23), and FEV_1%_/VC_max_ from 50 to 121 (*M* = 86.94, SD = 14.87).

In our sample, BMI ranged from 16.90 to 50.40 (*M* = 27.80, SD = 5.50). Detailed characteristics regarding smoking status and BMI in the sample are presented in Table 1.

**Table 1:** Smoking status and BMI in the study sample.

Smoking status	*N* (male/female)	Number of cigarettes smoked per day	Number of years smoking M (SD)	Pack years of smoking	Time since quitting	FEV_1%_	VC	FEV_1%_/VC_max_
		M (SD)		M (SD)	M (SD)	M (SD)	M (SD)	M (SD)
Never-smokers	27 (3/24)	–	–	–	–	89.52 (23.04)	96.93 (18.18)	94.85 (11.41)
Ex-smokers	56 (36/20)	21.87 (10.70)	29.47 (15.52)	35.19 (29.06)	16.51 (12.44)	70.64 (21.03)	85.16 (20.39)	83.57 (14.75)
Smokers	20 (6/14)	11.52 (5.06)	38.81 (23.03)	23.00 (13.01)	–	70.70 (27.32)	81.55 (22.39)	86.45 (16.50)

**Classification**		**BMI (kg/m^2^)**		**N (M/F)**		**FEV_1%_, M (SD)**	**VC, M (SD)**	**FEV_1%_/VC_max_, M (SD)**

Underweight		<18.50		2 (0/2)		41.5 (17.68)	68 (27.04)	62 (4.24)
Normal range		18.5–24.99		28 (12/16)		77.64 (25.96)	90.29 (22.37)	86.68 (15.54)
Pre-obese		25–29.99		46 (24/22)		73.76 (23.84)	85.63 (22.62)	86.87 (14.31)
Obese		≥30		29 (11/18)		76.55 (23.17)	86.86 (17.59)	89.03 (14.48)

*M, mean; SD, standard deviation.*

## Results

Patients with various smoking statuses were compared with respect to QoL and acceptance of life with illness while controlling for the effects of covariates. The results of this analysis are presented in Table 2.

**Table 2:** Differences between groups distinguished on the basis of smoking status.

HRQoL and ALDS	Never-smokers		Ex-smokers		Smokers		*F*	*p*	Effect sizes	
	*M*	SD	*M*	SD	*M*	SD			$\eta_{p}^{2}$	Cohen’s *d*
Cough	1.96	1.29	2.19	1.47	3.05	1.28	2.519	0.004	0.308	1.334
Expectoration	1.70	1.51	2.39	1.55	2.65	1.35	1.338	0.198	0.191	0.972
Chest tightness	1.30	1.51	1.78	1.46	1.95	1.82	1.710	0.064	0.232	1.099
Dyspnea	2.74	1.61	3.56	1.38	3.80	1.58	2.395	0.006	0.297	1.3
Limitation of daily activities	1.78	1.60	2.26	1.64	2.70	1.78	2.951	0.001	0.342	1.442
Lack of self-confidence	1.11	1.55	1.46	1.59	1.40	1.64	3.439	0.000	0.378	1.559
Quality of sleep	1.59	1.65	2.41	1.76	1.70	1.81	2.143	0.015	0.274	1.229
Energy levels	1.56	1.55	2.09	1.66	1.90	1.55	1.083	0.384	0.160	0.873
CAT global score	13.74	8.02	18.13	8.03	19.15	9.18	4.140	0.000	0.422	1.709
Satisfaction with life despite the disease	31.56	5.07	31.07	5.64	29.80	5.51	1.002	0.461	0.150	0.840
Reconcilement with the disease	21.41	3.44	21.46	3.38	20.95	3.03	0.755	0.722	0.118	0.731
Self-distancing from the disease	15.11	3.49	15.04	3.56	14.05	4.12	0.524	0.920	0.085	0.610
ALDS global score	68.07	11.06	67.56	11.33	64.80	11.34	0.713	0.765	0.112	0.710

The groups were found to differ statistically significantly in terms of Cough, Dyspnea, Limitation of daily activities, Lack of self-confidence, Quality of sleep, and CAT global score. The size of the effect ranged from intermediate (Reconcilement with the Disease, Self-distancing from the Disease, ALDS global score) to large (CAT dimensions and global score, Satisfaction with Life Despite the Disease). Further analyses showed that smokers differed statistically significantly from ex-smokers in Cough (*F* = 1.882; *p* = 0.047; ηp^2^ = 0.309; Cohen’s *d* = 1.337), Limitations of daily activities (*F* = 2.779; *p* = 0.003; ηp^2^ = 0.397; Cohen’s *d* = 1.623), Lack of self-confidence (*F* = 4.052; *p* < 0.001; ηp^2^ = 0.490; Cohen’s *d* = 1.960), Quality of sleep (*F* = 2.030; *p* = 0.031; ηp^2^ = 0.325; Cohen’s *d* = 1.388), and CAT global score (*F* = 3.507; *p* < 0.001; ηp^2^ = 0.454; Cohen’s *d* = 1.824). Smokers also differed statistically significantly from never-smokers in Cough (*F* = 2.538; *p* = 0.014; ηp^2^ = 0.526; Cohen’s *d* = 2.107), Dyspnea (*F* = 2.581; *p* = 0.013; ηp^2^ = 0.530; Cohen’s *d* = 2.124), and CAT global score (*F* = 2.641; *p* = 0.011; ηp^2^ = 0.536; Cohen’s *d* = 2.150). Never-smokers differed statistically significantly from ex-smokers in Dyspnea (*F* = 1.878; *p* = 0.045; ηp^2^ = 0.285; Cohen’s *d* = 1.263), Limitations of daily activities (*F* = 3.179; *p* < 0.001; ηp^2^ = 0.403; Cohen’s *d* = 1.643), Lack of self-confidence (*F* = 3.176; *p* < 0.001; ηp^2^ = 0.403; Cohen’s *d* = 1.643), Quality of sleep (*F* = 1.950; *p* = 0.036; ηp^2^ = 0.293; Cohen’s *d* = 1.288), and CAT global score (*F* = 3.404; *p* < 0.001; ηp^2^ = 0.419; Cohen’s *d* = 1.698).

In the next step, analysis of covariance between the subgroups of obese (BMI > 30) and normal weight participants (BMI: 18.5–24.99) was conducted (Table 3).

**Table 3:** Differences between the groups divided according to BMI.

HRQoL and ALDS	Normal-weight		Obese		*F*	*p*	Effect sizes	
	*M*	SD	*M*	SD			ηp^2^	Cohen’s *d*
Cough	2.44	1.28	2.17	1.31	2.451	0.013	0.456	1.831
Expectoration	2.52	1.60	1.93	1.22	1.032	0.443	0.261	1.189
Chest tightness	1.89	1.67	1.86	1.55	1.515	0.149	0.341	1.439
Dyspnea	3.26	1.56	3.52	1.30	1.956	0.048	0.400	1.633
Limitation of daily activities	2.19	1.71	2.55	1.45	3.626	0.001	0.553	2.225
Lack of self-confidence	1.52	1.70	1.24	1.50	3.816	0.000	0.566	2.284
Quality of sleep	2.11	1.83	2.55	1.66	1.242	0.284	0.298	1.303
Energy levels	1.81	1.59	2.07	1.58	0.956	0.512	0.246	1.142
CAT global score	17.74	8.85	17.90	7.27	3.118	0.002	0.516	2.065
Satisfaction with life despite the disease	31.93	4.57	29.57	6.65	0.833	0.631	0.221	1.065
Reconcilement with the disease	21.52	3.70	20.95	3.37	0.690	0.770	0.191	0.972
Self-distancing from the disease	14.70	3.23	15.00	3.63	0.693	0.768	0.191	0.972
ALDS global score	68.15	10.75	65.52	12.59	0.748	0.715	0.203	1.010

These subgroups differed statistically significantly in Cough, Dyspnea, Limitation of daily activities, Lack of self-confidence, and CAT global score. For all QoL dimensions and for Acceptance, the effect sizes were large.

The analysis of partial correlations between dimensions of QoL (as measured by CAT) and Acceptance (as measured by ALDS) showed only negative interdependence between Limitations in daily activities and Self-distancing from the Disease (*r* = -0.225; *p* = 0.033).

## Discussion

In our study we aimed to identify the relationship of BMI and smoking status with individual dimensions of QoL in patients with COPD, especially after controlling for possible confounding effects of sociodemographic variables. Our results showed that smoking status remains negatively related to five aspects of QoL (Cough, Dyspnea, Limitation of daily activities, Lack of self-confidence, Quality of sleep) in such a way that never-smokers had better QoL than smokers and ex-smokers, and the latter two subgroups did not differ in terms of QoL dimensions. This remained true even after controlling for the effect of sex, age, marital status, place of residence, education, professional activity, years since COPD diagnosis, number of hospitalizations due to COPD, subjective self-assessment of COPD severity and progression, and airflow obstruction indicators. Further analyses found that people who have smoked in the past are characterized by the highest coughing severity and the lowest QoL associated with the disease, operationalized as CAT global score. People who have never smoked have the lowest degree of dyspnea and the highest QoL (as measured by the CAT global score). This finding corroborates the view that even complete cessation of smoking does not guarantee the complete elimination of the adverse effects of smoking (Hodge et al., 2005; Louhelainen et al., 2009), and these effects are also influenced by additional factors such as the stage of disease and general physical and mental health. For instance, other analyses show that severity of depressive symptoms explains about 40% of variance in QoL as measured by CAT (Miravitlles et al., 2018). It is known that irreversible changes occur in the respiratory system in the course of COPD (Global Initiative for Chronic Obstructive Lung Disease, 2018), and our analyses suggest that current smoking and ex-smoking status is an independent factor that contributes to lower QoL through exacerbation of COPD symptoms.

After controlling for the effect of potential covariates, our patients who qualified as obese (based on their BMI) showed significantly greater impairment of QoL in dyspnea, limitations in everyday activities, and the global index of QoL. Interestingly, patients with obesity had better QoL with respect to self-confidence and cough. These findings provide an argument for the claim that the relationship between obesity and QoL in COPD is complex and probably shows variation with respect to particular QoL dimensions. Previous research indicated reduced mortality (Landbo et al., 1999; Spelta et al., 2018) and less severe airflow obstruction (Cecere et al., 2011; Divo et al., 2014) among obese patients, a phenomenon known as the COPD-obesity paradox. It has been suggested that obese patients may receive more intense in-patient treatment, including mechanical ventilation, and may stay in the hospital longer (due to obesity-related comorbidities), despite comparable (or perhaps even less severe) airflow limitation. On the other hand, higher BMI may be related to differences in body composition including greater muscle mass, which predicts better outcomes in COPD to a greater extent than adiposity (McAuley and Beavers, 2014). It may also be possible that the COPD-obesity paradox is observed mostly in hospitalized patients in contrast to outpatients with a low rate of COPD-related hospitalization and stable airflow obstruction indicators.

We decided to include both positive (Acceptance of Life with Illness) and negative (CAT) QoL indicators. Fewer studies have to date have focused on positive indicators of adaptation to life with chronic diseases such as COPD. It seems important that negative indicators of adaptation, such as psychopathological symptoms or the degree to which the disease reduces QoL, do not provide a legitimate way to draw sufficiently justified conclusions regarding optimal adaptation. Optimal adaptation, especially optimal psychological adaptation to life with chronic disease, means not only a lack of psychopathological or disease symptoms, but also the presence of positive mental states, such as a sense of life satisfaction, commitment to achieving goals, positive social relationships, etc. Meanwhile, such positive indicators of adaptation are missing from many studies on stress and COPD.

Our study did not reveal statistically significant differences in acceptance of life with illness between patients with various smoking or BMI statuses. However, the magnitudes of the effects sizes ($\eta_{p}^{2}$, Cohen’s *d*) ranged from intermediate to large, which suggests a clinically significant difference (Cohen, 1988). Also, the analyses of partial correlations showed that QoL, as assessed by CAT, was not related to level of acceptance of life with illness. This may be due to the fact that acceptance is a psychological construct, more permanent and not necessarily sensitive to the severity of symptoms. In addition, the relationship between acceptance and smoking and BMI status may be mediated by other variables, such as the severity of depressive symptoms (Ng et al., 2007).

In conclusion, the results of our study suggest a certain path for the potential effects of obesity and smoking status on QoL. Risky health behaviors, such as smoking and at-risk body mass, only affect some aspects of health-related QoL – specifically cough, dyspnea, and limitations in everyday life functioning. This suggests that severity of cough and dyspnea may be directly affected by these two lifestyle factors, and this, in turn, may be a key factor affecting limitations in everyday life functioning and self-reported quality of sleep, as measured by CAT.

### Limitations and Directions for Future Research

This study also has some limitations. It only included a relatively small number of patients. There is a need to conduct prospective studies on a larger sample. Also, our study did not take into account data concerning patients’ comorbidities and pharmacotherapy doses. For example, in the subgroup of obese patients, the doses used in therapy may be larger than in normal and underweight patients, regardless of the severity of the airflow obstruction.

Future studies should also take into consideration different operationalizations of smoking habits. More specific data could be gathered, such as the duration of periods of abstinence and differences in the number of cigarettes smoked per day. In our opinion, the pack-year index may not be the best description and does not fully reflect the characteristics and dynamics of changes in the course of smoking addiction. Since previous studies have indicated that severity of depressive and anxiety symptoms are very important factors associated with both QoL and clinical features of COPD (Ng et al., 2007; Mathews et al., 2018; Miravitlles et al., 2018), future research should take into account the presence of these psychopathological symptoms as covariates when elucidating the effects of BMI and/or smoking status on QoL in patients with COPD. In future studies it would also be worth taking into account more complex indicators of body mass, e.g., using ultrasonography or computer tomography to determine exact body composition.

## Conclusion

– Both smoking status and BMI are associated with certain dimensions of QoL in stable outpatients with COPD.
– Smoking, both in the past and currently, decreases QoL independently of sociodemographic and clinical variables.
– Obesity is ambiguously related to QoL, with some dimensions of QoL being higher and others lower in patients with obesity compared to normal-weight patients.

## Data Availability

The datasets for this study will not be made publicly available because we do not have permission from participants to share their medical and psychological data. According to the GDPR, we have to get permission from the participants to use their data only in a particular way.

## Ethics Statement

This study was conducted according to the guidelines of the Declaration of Helsinki. The approval of the Bioethical Commission of the University of Economics and Human Sciences in Warsaw was obtained for this study and all subjects gave their written informed consent prior to their participation in the study.

## Author Contributions

KW, DP, KJ, KB-M, and AF wrote the manuscript. KW, DP, and JD conducted the research. KW and DP analyzed the data.

## Conflict of Interest Statement

The authors declare that the research was conducted in the absence of any commercial or financial relationships that could be construed as a potential conflict of interest.
